# Ocular Complications of Diabetes and Therapeutic Approaches

**DOI:** 10.1155/2016/3801570

**Published:** 2016-03-28

**Authors:** Victoria J. Vieira-Potter, Dimitrios Karamichos, Darren J. Lee

**Affiliations:** ^1^Department of Nutrition & Exercise Physiology, University of Missouri, Columbia, MO 65211, USA; ^2^Department of Cell Biology, Dean McGee Eye Research Institute, University of Oklahoma Health Sciences Center, Oklahoma City, OK 73104, USA; ^3^Department of Ophthalmology, Dean McGee Eye Research Institute, University of Oklahoma Health Sciences Center, Oklahoma City, OK 73104, USA; ^4^Department of Microbiology and Immunology, Dean McGee Eye Research Institute, University of Oklahoma Health Sciences Center, Oklahoma City, OK 73104, USA

## Abstract

Diabetes mellitus (DM) is a metabolic disease defined by elevated blood glucose (BG). DM is a global epidemic and the prevalence is anticipated to continue to increase. The ocular complications of DM negatively impact the quality of life and carry an extremely high economic burden. While systemic control of BG can slow the ocular complications they cannot stop them, especially if clinical symptoms are already present. With the advances in biodegradable polymers, implantable ocular devices can slowly release medication to stop, and in some cases reverse, diabetic complications in the eye. In this review we discuss the ocular complications associated with DM, the treatments available with a focus on localized treatments, and what promising treatments are on the horizon.

## 1. Introduction

We are experiencing a worldwide increase in the prevalence of diabetes mellitus (DM) (i.e., diabetes), a group of metabolic diseases characterized by chronically elevated blood glucose levels. DM is further classified as type 1 (T1DM), which results from pancreatic beta cell failure such that insufficient insulin is produced to effectively clear blood glucose; type 2 (T2DM), which is defined by a state of insulin resistance whereby target cells fail to effectively respond to the hormone, insulin; and gestational DM, which occurs when pregnant women develop insulin resistance during pregnancy. In 2013, an estimated 382 million people were diagnosed with diabetes with T2DM accounting for 90% of the cases [1].

The public health burden of DM is largely attributed to the fact that hyperglycemia increases the likelihood of both macrovascular and microvascular complications; indeed, it is these degenerative complications that result in the increase in morbidity and mortality associated with all forms of DM [2]. When not properly managed, long-term complications of this group of diseases can be severe and include heart disease, stroke, and kidney failure. Importantly, diabetes also profoundly impacts the ocular tissue, with damage to this organ occurring even at the early stages of the disease. While the most prominent manifestation of impaired macrovascular function in DM is accelerated atherosclerosis, microvascular dysfunction leads to nephropathy and retinopathy [2]. Among the microvascular complications of diabetes, diabetic retinopathy (DR) is the most common and is the leading cause of blindness among working-age adults in Westernized societies [3]. Mechanistically, the changes in the microvasculature result in increased vascular permeability and ischemia [4]. The most profound effects of these alterations are seen in the cornea and retina of the eye.

The cause of T1DM is uncertain and it is not preventable, while T2DM is almost always preventable via behavioral approaches such as diet, exercise, and weight control [1]. Even when well controlled, diabetes has a profound adverse effect on the ocular tissues, which parallels the severity of the disease and the stage at which it was diagnosed. When DR becomes chronic, corneal impairments are almost inevitable. Once the eye has been exposed to hyperglycemia long-term, the basement membrane has accumulated enough toxic end products to lead to cell death, opacity, and eventually vision impairment [5–9], which is irreversible. Although the most common, DR is not the only ocular complication of diabetes; others include corneal dysfunction, cataract, glaucoma, neuropathy, ischemic optic neuropathy, and diabetic macular edema (DME) [1, 10]. Several of these are candidate conditions for therapeutic approaches utilizing tissue engineering.

## 2. Ocular Complications Associated with Diabetes

### 2.1. Diabetic Retinopathy (DR)

DR is a progressive blinding disease that affects 4.2 million people worldwide, making it a leading cause of blindness; and, this number is expected to continue to increase [10–13]. DR can be divided into two types, nonproliferative DR (NPDR) and proliferative DR (PDR). NPDR can be further divided into three stages before progressing to PDR (Table 1). An important difference between NPDR and PDR is that vision is not compromised with NDPR, whereas PDR is vision threatening. While NPDR almost always progresses to PDR, the progression can be delayed with tight blood glucose control [14].

The etiology of DR is complex and not completely understood. However, the mechanisms likely involve vascular, neuronal, and immunological systems [3]. The visual cycle puts a high metabolic demand on the retina, which has two sources of vascular supply. Retinal arteriole vessels supply 2/3 of the inner retina, while the choroid supplies the retinal pigmented epithelial cells and the outer 1/3 of the retina [15]. One of the earliest changes that occur in DR is a reduction in retinal perfusion. These microvascular changes are not always apparent to the patient but are visible on a fundus examination. The reduced blood supply triggers a series of adverse metabolic reactions that ultimately result in endothelial cell degeneration of the retina. The result is retinal ischemia, increased compensatory angiogenesis, tissue remodeling, and inflammation characterized by increased expression of VEGF, IL-6, IL-1*β*, and TNF-*α* [3]. Retinal vessels are particularly susceptible to the microvascular changes that are associated with hyperglycemia [12, 13]. Mechanistically, there are numerous biochemical pathways that link hyperglycemia to the reduced vascularization that is intrinsic to the pathology of DR. Some of these include polyol accumulation, oxidative stress, increased expression of angiogenic factors, and activation of protein kinase C [3, 16, 17]. Moreover, hyperglycemic conditions may directly impair retinal mitochondria resulting in increased ROS, inflammation, and DNA damage [18]. Together, microvascular changes, reduced perfusion, thickening of the basement membrane, and systemic abnormalities, such as hypertension, converge to cause retinal pericyte loss, ultimately leading to neovascularization [19, 20]. Once this pathological cycle begins, controlling blood glucose has little or no effect on the ocular diabetic complications. That is largely due to a cascade of inflammatory and angiogenic factors that no longer respond to well controlled blood glucose levels. Therefore, DR likely will eventually require implant therapy.

### 2.2. NPDR

The first stage of NPDR is mild NPDR in which microvascular changes manifest as microaneurysms that are visible on the retina. NPDR is classified as moderate when intraretinal hemorrhages, hard exudates, cotton wool spots, and venous beading in two or less quadrants are visible on the retina. The intraretinal hemorrhages usually clear up in two to three weeks and so do not interfere with vision long-term. Severe NPDR occurs as the duration of disease continues, the intraretinal hemorrhages increase to include all four quadrants, venous beading increases to include more than two quadrants, and/or one intraretinal microvascular abnormality is visible.

### 2.3. PDR

The microvascular changes result in a constriction of the blood vessels that nourish the retina [21]. In response to the reduction of retinal perfusion and retinal hemorrhage, abnormal growth of new retina blood vessels occurs. These vessels are problematic because red blood cells absorb light to obscure vision. This neovascularization marks a critical distinction between NPDR and PDR. These vessels can grow into the vitreous and if left untreated can result in retinal tearing and detachment [22]. Additionally, the walls of these abnormal vessels are susceptible to breakage, resulting in vitreous hemorrhage that causes two additional problems. First, the blood from vitreous hemorrhage impairs vision. Retinal vessels have a blood retinal barrier that prevents plasma, growth factors, and other inflammatory factors from entering the immunologically quiescent eye. Therefore, a second consequence of vitreous hemorrhage is that it triggers additional neovascularization and inflammation that perpetuates PDR [4].

### 2.4. Diabetic Macular Edema (DME)

The macula is located in the center of the retina and contains the highest concentration of cones. This gives the ability to see color and details. Because of the central location of the macula, it also means the macula is responsible for central vision. When the fragile retinal vessels burst, the fluid accumulates causing a thickening of the retina. This results in distorted or blurry vision [23]. It has been observed that the incidence of DME is higher among type 2 compared to type 1 diabetics, but it is not well understood why this is the case [24].

### 2.5. Neuropathy

Visual information is transmitted to the brain as electrochemical signals through retinal ganglion cells (RGCs). The RGCs bundle together to form the optic nerve that exits the back of the eye and transfers the visual information to the brain. Diabetes causes stress to the retina that triggers apoptosis of RGCs [25, 26]. These stresses include ischemia, oxidative stress, a reduction of trophic factors, excitotoxicity, increased intraocular pressure, neuroinflammation, and aldose reductase inhibition. Unfortunately, RGC death can occur even during the early stages of diabetes [27–29].

### 2.6. Ischemic Optic Neuropathy

The reduced retinal perfusion and microaneurysms can reduce the oxygen supply to the optic nerve. If the optic nerve is deprived of oxygen for too long it will undergo apoptosis and cause permanent vision loss. The ischemia and neuropathy trigger a cascade that results in the release of additional apoptotic factors that can affect other areas of the retina. In addition the ischemia induces hypoxia inducible factors that promote angiogenesis and inflammation [30]. Therefore, the optic neuropathy that results from ischemia is just one result of the low oxygen conditions.

### 2.7. Glaucoma

The World Health Organization has classified glaucoma as priority eye disease [31]. When aqueous humor does not properly drain through the trabecular meshwork and Schlemm's canal, it can lead to excess pressure inside the eye. The increase in pressure can damage nerves and the blood vessels, causing changes in vision, leading to glaucoma. It is projected to affect 79.6 million people by 2020 [32]. However, the studies documenting the magnitude of glaucoma among diabetics worldwide are limited. Its prevalence among diabetics ranges from 4.96% to 14.6% [33, 34]. Two main categories exist: “open-angle” and “closed-angle” glaucoma. Open-angle glaucoma is painless, chronic, and generally asymptomatic until it progresses significantly. Closed-angle glaucoma, on the other hand, is known for sudden eye pain, redness, nausea, and intraocular pressure spikes. While open-angle glaucoma can be treated with medications, closed-angle glaucoma generally requires medical emergency care. People with diabetes tend to get open-angle glaucoma more than closed-angle glaucoma. However, there is no study showing an increased rate of primary open-angle glaucoma in diabetics. Four of the major studies in the last twenty years cannot agree largely due to inconsistent definitions of both DM and glaucoma and study exclusions or sampling bias. The Beaver Dam Eye Study (1994) showed diabetics (mostly T2DM) with glaucoma incidence of 4.2% versus 2.0% in participants without DM. When people treated for glaucoma were included, rates were 7.8% in diabetics compared with 3.9% in those without diabetes. A year later, 1995, the Baltimore Eye Survey concluded that diabetics are no more likely to have open-angle glaucoma than nondiabetics. DM was defined in this study based on history only. The authors suggest that previous reported increase in prevalence is due to more screening in diabetics. The Rotterdam Study (1996) reported that newly diagnosed diabetics had increased prevalence of open-angle glaucoma [35, 36]. In 2002, the Ocular Hypertension Treatment Study showed protective effect of DM on open-angle glaucoma. That study excluded patients with DM. People with diabetes are also more likely to get an uncommon type of glaucoma, called neovascular glaucoma. In this form of glaucoma, new blood vessels grow on the iris. These blood vessels block the normal flow of fluid out of the eye, raising the eye pressure. This particular type is very difficult to treat. One option is laser surgery to reduce the vessels. Surgeons are also looking into the use of implants to help drain the fluid. Injectable anti-VEGF medications are also widely used [37].

### 2.8. Corneal Edema

Common corneal dysfunctions associated with diabetes result in impaired vision or blindness due to decreased wound healing, corneal edema, and an altered epithelial basement membrane. In fact, of the 382 million people diagnosed with DM worldwide, approximately 70% suffer from some kind of corneal complications collectively and commonly known as diabetic keratopathy [5, 38–41]. Corneal repair is often difficult in diabetic patients. The diabetic cornea suffers from cellular dysfunction and dysfunctional wound healing/repair mechanisms [42–45]. There have been an extensive range of studies looking at specific dysfunctions of the cornea. Schultz and coauthors [40] found corneal epithelial lesions in more than 65% of the population tested. Two years later [46], the same group reported diminished corneal peripheral sensation suggesting some kind of neuropathy. This has now been confirmed by multiple studies [40, 47–53] and is widely accepted that these patients suffer from reduced corneal sensitivity and generalized neuropathy. Diabetic patients have also been found to have abnormal adhesions of the corneal epithelium to the underlying basement membrane [6] leading to prolonged and recurrent defects. To make things even more complicated, Gekka et al. [54] and Göbbels et al. [55] showed improper function and weakening of the epithelial barrier in diabetic patients leading to higher risks of corneal infections and stromal fibrosis. Corneal thickness increase has also been reported [56–59] and linked to diabetes as well as endothelial dysfunction [58]. Clearly, there are a lot of defects in the human diabetic cornea that may lead to severe vision impairments.

### 2.9. Corneal Nerve Alterations

Diabetes-related microvascular complications include, but are not limited to, nephropathy, end-stage renal failure, peripheral neuropathy, and blindness [68]. The prevalence of these complications is highly dependent upon disease duration and age. Recent technological advancements have enhanced our ability to monitor and diagnose ocular diabetes defects.* In vivo* confocal microscopy has played a critical role in defining the role of corneal subbasal nerves in diabetes [69–72]. Rosenberg and coauthors showed a correlation between corneal subbasal nerve density and corneal sensation in a group of 23 patients diagnosed with type I diabetes [53]. Edwards et al. [73] recently used an automated imaging technique to compare montages of the subbasal nerve plexus between healthy and diabetic patients. They mapped the entire human corneal nerve architecture and demonstrated that epithelial nerve density of the central cornea is higher than that in the periphery [73]. Intriguingly, the reduced subbasal nerve density in the cornea has been associated with diabetic retinopathy and peripheral neuropathy [74–77]. Reduced corneal sensitivity resulting from defected subbasal nerves has also been noted as a potential biomarker for autonomic cardiac neuropathy (a diabetic complication) [65]. Overall, diabetes can severely affect the ocular surface as well as other ocular structures such as the retina.  Table 2 lists the major clinical defects observed in patients with early, mild, and end-stage diabetes with regard to the cornea, retina, and nerves.

### 2.10. Cataract

Cataract is one of the main causes of vision impairment in diabetics. Although cataract surgery is relatively safe and has high rates of success among healthy individuals, that is not the case with diabetics. Klein and coauthors reported a large proportion (59–98%) of people with T2DM aged 30 to 75 will develop cataract [78, 79]. Other studies have reported greater foveal thickness and higher incidence of macular edema following cataract surgery in diabetic compared to nondiabetic patients [80–82]. Posterior capsular opacification (PCO) is a common finding following cataract surgery. When the lens is removed during cataract surgery, the capsule that the lens sits in remains and in some cases it can obstruct vision by opacification. A higher incidence of PCO was reported in diabetics [83]. Zaczek and Zetterstrom, however, reported the exact opposite, where PCO rates were reduced in diabetics. Another area of conflicting reports is whether or not cataract surgery accelerates DR. There is evidence for both sides of that argument as reviewed by Skarbez et al. [38]. Some, but not all, report that DR is progressed following extracapsular cataract extraction [83–86]. As with DR, there are concerns that cataract surgery in diabetics may exacerbate the progression of macular edema. However, the studies available suggest that this is only of minor concern with the vast majority showing no evidence of macular edema in these patients [86].

## 3. Risks Involved in Common Ocular Procedures among Diabetics

### 3.1. Corneal Transplants

In the United States alone, more than 40,000 corneal transplants are performed annually [87]. Over the years, corneal transplantation success has risen mainly due to technological advances. However, despite the improved success rates, several problems can occur including rejection of the new cornea. About 20% of corneal transplants are rejected [87, 88]. There are several types or corneal transplants including full thickness, lamellar, Descemet's stripping automated endothelial (DSAEK), Descemet's membrane endothelial keratoplasty (DMEK), and anterior lamellar corneal transplants [89–92]. Depending on the location of the scars and the degree of corneal damage, the surgeon can make a decision on which of the above is more appropriate. In diabetes, there are several problems that occur and may lead to corneal transplant. Some of the most common corneal defects seen in diabetics are recurrent corneal erosions, persistent epithelial defects, and corneal endothelial damage. The long exposure to abnormal glucose levels can lead to blindness and corneal transplantation is, most of the time, the first treatment option. In diabetics, success of corneal transplantation is lower when compared to other diseases such as keratoconus. One of the main reasons for this is that diabetics have a slower wound healing process causing grafts to fail quicker. In addition, diabetics are more prone to corneal infections such as fungal keratitis which can also cause graft rejection [93].

### 3.2. LASIK/PRK

Laser* In Situ* Keratomileusis (LASIK) is a common corrective vision procedure for millions of people annually. Given the increased incidence of corneal defects in diabetic patients, several investigators are looking into the risks of LASIK performed in these patients. One of the first reports described poor refractive results and epithelial complications in 47% of diabetic patients [94]. Others have reported epithelial ingrowth following LASIK [95–98]. Another study by Ghanbari and Ahmadieh [99] described extensive neovascularization of the iris and rapid advancement of proliferative DR following LASIK, raising concerns about the link between LASIK and DR. The alternative to LASIK is photorefractive keratectomy (PRK). PRK involves removal of the epithelium which is problematic in diabetics where the epithelium is known to heal slowly. In that respect, LASIK may be preferred since it involves making a flap and applying the laser directly to the stroma; however trauma is still done to the epithelium. Despite the risks, both PRK and LASIK have enabled millions of people to achieve better vision. There are clearly pros and cons for either one of these techniques, but in the end the surgeon and the patients are the ones choosing the best option [100–104].

## 4. Treatment Options

### 4.1. Blood Glucose Control

The first line of defense in managing all forms of DM is tight blood glucose control; indeed, hyperglycemia is the main determinant of diabetic microvascular diseases [2]. Thus, pharmacological blood glucose control is often thought to be efficacious in preventing and treating DR. There are six classes of oral glucose-lowering drugs: biguanides (e.g., metformin), sulfonylureas (e.g., glimepiride), meglitinides (e.g., repaglinide), thiazolidinediones (e.g., pioglitazone), dipeptidyl peptidase IV inhibitors, and alpha glucosidase inhibitors (e.g., acarbose). When these oral glucose-regulating drugs are insufficient to stabilize blood glucose, insulin therapy is used. Sulfonylureas are the oldest and most widely used drug class. While they are effective glucose-lowering agents, they may cause hypoglycemia and weight gain. Metformin is an insulin-sensitizing drug, so it is only effective prior to beta cell failure. It is also particularly effective at reducing inflammation and endothelial dysfunction associated with T2DM, making it an attractive therapeutic approach for DR.

### 4.2. Insulin Eye Drops

Some diabetics are required to use insulin injections in order to control blood sugar levels. However, despite the popularity of this method many patients find it difficult to maintain correct sugar levels. One of the alternative methods of delivering insulin that have been studied is insulin eye drops. In animal studies, applying insulin eye drops has been relatively effective [105]. Pillion et al. investigated the efficacy of insulin eye drops in rats at a 2 mg/mL concentration and found that they could not be absorbed when delivered in saline [106]. However, the absorption was massively increased when the authors added various emulsant agents such as saponin, Brij-78, and BL-9. In a more recent study, Liu and colleagues determined the efficacy of insulin eye drops in rabbit eyes using Brij-78 as a delivery agent [107, 108]. Absorption was found optimum at 0.05% insulin and 0.5% Brij-78. Concentrations were extrapolated for future human studies and suggested a therapeutic dose of 1.25% insulin or 1.25 mg insulin/75 kg body weight. Overall, it is easily understood that delivering insulin through the ocular route is much easier and less expensive than injections.

### 4.3. Blood Sugar Contact Lenses

A more sophisticated and technologically advanced method for measuring and controlling sugar levels was recently developed by Otis and Parviz [109]. They have created contact lenses that can detect and measure blood sugar levels in human tears throughout the day. A thin glucose-sensing chip is sandwiched between two layers of soft contact lens material, with a small pore over the sensor. The eye naturally generates tears over the course of the day to keep the eye lubricated. The tears leak into the pore, reach the sensor, and transmit the reading wirelessly to an external device (e.g., smartphone). Despite the very promising technology, there are major hurdles to overcome before these sensors are commercially available. First, they have to meet the consumers' criteria of acceptance including convenience, wear schedule, cleaning, and cosmetics. The other major factor, in today's hi-tech world, is powering the contacts lenses. To date, wireless powering is possible using electromagnetic radiation at high frequency which has significant health effects. Even if we assume that all the above are possible and consumers are ready for such a product, the supplier will have to gain marketing approval and follow the governing regulations which may vary substantially between countries. In the USA, as recently reviewed by Farandos et al., the FDA has three regulatory classes for medical devices. Class I is associated with the lowest risk and is least regulated and Class III the highest risk and most regulated [105]. Overall, the technology is not market-ready yet. Google's glucose lenses have been recently licensed by Novartis and give an opportunity for further development and commercialization.

### 4.4. Subconjunctival Glucose Sensors

There is enough evidence that tight glycemic control is mandatory to prevent, or at the very least slow, the progression of chronic complications such as blindness [110]. In 2012, Müller and coauthors developed a long-term implantable glucose sensor which was an upgrade to the* in vitro* blood glucose test strips and the short-term sensor implants currently available for blood glucose monitoring [111]. The novel long-term blood glucose monitoring system is an ocular mini implant placed under the bulbar conjunctiva of the patient's eye and a handheld fluorescence photometer reads out the sensor signal from the implant and translates it to a blood glucose reading. The study showed no toxicity. The functional components are dye-labeled Con A and dextran, which are known to be safe at low doses. The system has only been tested for two weeks* in vivo*. Longer studies are clearly needed. One of the major drawbacks is that the glucose measurements are self-performed and the photometer positioning has to be precise and accurate in order to ensure correct measurements. Müller and coauthors published a long-term study in 2013 where they evaluated the implantable subconjunctival glucose monitoring system (SGMS) for long-term glucose monitoring [112]. SGMS, a modified version of the original implantable device, contains a proprietary hydrophilic biocompatible surface coating in order to minimize fibrosis seen with the original uncoated design. They performed a 1-year clinical study with 47 diabetes patients. While the results were promising, further design modifications are required before a final product can be developed. Two major problems were noted. The first was that the device showed decreased measurement performance, as fibrous tissue was forming around the implant. Second, the two-point calibration system used may not be applicable for a final product where real time glucose displayed values are necessary.

### 4.5. Management and Treatment Options for Corneal Edema

Corneal edema/scarring management and treatment are currently identical, whether the edema is a result of an injury, trauma, or disease. The options available to patients with associated symptoms have been previously described and reviewed [113–120]. In the following section, we will briefly discuss edema management and clinical treatment options.

Patients with mild corneal edema are normally prescribed hypertonic agents, such as sodium chloride 2% and 5% solution and ointment. These agents create a hypertonic tear film that draws water out of the cornea limiting the buildup of edema.

Bandage contact lenses are also available for temporary relief of corneal pain and discomfort [121, 122]. These contact lenses prevent the cornea from coming into contact with the eyelids which can be painful due to the corneal injury and the damaged epithelium. However, there are several concerns with contact lenses in general. Improper or overnight wear can actually lead to more corneal edema as well as an increased risk of infection. For these reasons, bandage contact lenses are for short-term early treatment, are prescribed with antibiotics, and require close follow-up medical care.

For patients with severe pain, anterior stromal puncture is performed [123]. Normally, a 25-gauge needle is used to deliver multiple superficial punctures just below Bowman's layer. This technique normally leads to a strengthened bond between the epithelium and Bowman's layers. Similar results have been achieved using laser phototherapeutic keratectomy.

Ultimately, when the vision decreases significantly by corneal edema the one definitive treatment is corneal transplantation. Corneal transplantation can be performed in several ways depending on the location of the pathology in the host cornea. This topic is extensive and there are multiple reviews that outline techniques, advantages, and disadvantages. While this is beyond the scope of this review, we outline a few of the most important and widely used corneal transplantation techniques below.

Full thickness corneal transplantation or penetrating keratoplasty (PKP) is one of the original and most common techniques where the full thickness tissue is removed and replaced by a donor tissue [124]. PRK's major advantage is the minimization of tissue interfaces in the visual axes which ensure optical clarity. Disadvantages include postoperative wound leak and intraoperative hemorrhage.

Deep anterior lamellar keratoplasty (DALK) is an alternative to PKP when the host endothelium layer is still functional and the pathology is located within the anterior cornea [125–128]. Briefly, the host epithelium and stroma are removed and replaced by the donor corneal graft consisting of the epithelium, Bowman's membrane, and the corneal stroma. The major advantage is the retention of the host endothelium and Descemet membrane. On the other hand, one of the major risks is that if the host stromal layer is not removed completely an irregular stroma-to-stroma interface may form with the donor tissue.

Descemet stripping automated endothelial keratoplasty (DSAEK) is the technique commonly used for endothelial dysfunction and disease [129–135]. Briefly, the diseased host corneal endothelium is removed together with Descemet membrane and replaced by the donor endothelium, Descemet membrane, and some posterior stroma. Although DSEAK is a relatively new technique and survival graft survival data is not great, 3-year survival seems to be an accurate estimate. However, major complications with DSAEK can occur including endothelial rejection, primary graft failure, and iatrogenic glaucoma.

### 4.6. Antisteroidal Implants

Inflammation has a role in the ocular complications associated with diabetes [136]. Moreover, anti-inflammatory treatments have been shown to slow the progression of ocular complications. Because of the location and anatomy of the eye, drug delivery to the retina is difficult. One option is an intravitreal injection. However, because of the need for repeat injections, intraocular implants that deliver corticosteroids have been developed. Intraocular implants can be completely biodegradable or the biodegradable polymer can be encased in another polymer that is nonbiodegradable. Biodegradable implants are not anchored to the sclera, so they can move around and obscure vision. It has been reported that the polymer migrated to the anterior chamber to contact the corneal endothelium [137, 138]. This is problematic because it can erode through the cornea. Nonbiodegradable implants have the benefit that they may be anchored to the sclera to prevent migration. However, some patients may require removal of the implant once the medication has been exhausted [139, 140].

### 4.7. Ozurdex®

Ozurdex (Allergan Inc., Irvine, CA) delivers dexamethasone for up to 4 months and is completely biodegradable [141]. Ozurdex can be delivered with a designer applicator in a clinical setting and an IOP increase is less likely with this implant [142–145]. A ≥ 15 letter gain at 36 months was achieved in 22% of patients in the Macular Edema: Assessment of Implantable Dexamethasone in Diabetes (MEAD) Study, which is significantly greater than 12% observed in the sham injected group [146–148].

### 4.8. Retisert®

Retisert (Bausch & Lomb, Rochester, NY) is a nonbiodegradable implant that delivers fluocinolone acetonide for up to 30 months [141]. Delivery of Retisert involves an outpatient surgical procedure so that the implant can be anchored to the sclera [149]. While Retisert is not FDA approved for the treatment of DME, the effectiveness of Retisert was compared with laser treatment alone [150]. At 6 and 24 months the implant group showed significantly more patients with an improvement in visual acuity compared to the laser group. However, at 36 months there was no significant difference between the two groups [150]. The authors suggest that the lack of significance could be due to a decrease in drug availability. It should also be noted that the number of patients at 36 months is almost half the number at 6 months which would skew the dataset since it is likely the patients with improved vision would be less likely to be included in the 36-month time point.

### 4.9. Iluvien®

Iluvien (Alimera Science, Alpharetta, GA) delivers fluocinolone acetonide within a nonbiodegradable tube for up to 36 months [141]. The delivery of Iluvien can be accomplished with injection with a 25-gauge needle that can be performed in a clinical setting. In the Fluocinolone Acetonide in Diabetic Macular Edema (FAME) A and B studies it was found that a ≥ 15 letter gain was significantly greater at 34% compared to 13.4% in the sham group at 36 months [151].

### 4.10. Antineovascular Treatment

Since neovascularization and inflammation perpetuate one another, neutralization of inflammatory factors can also stop neovascularization and by inhibiting neovascularization the inflammation can be controlled as well. VEGF is a key signaling molecule that stimulates neovascularization. Therefore, another target to treat ocular diabetic disease is VEGF. There are currently four anti-VEGF treatments available and these are most effectively delivered to the retina through repeat intraocular injections.

### 4.11. Pegaptanib

Pegaptanib (Macugen, Pfizer, New York, NY) was approved for treatment of age-related macular degeneration in December 2004. Pegaptanib is an RNA aptamer that targets the VEFG-165 isoform. In double-masked clinical trials pegaptanib was effective in improving vision and reducing diabetic macular edema compared to laser therapy alone [152, 153]. However, pegaptanib has become eclipsed more recently because of the increased efficacy of the pan-VEGF inhibitors, ranibizumab and bevacizumab [154], discussed below.

### 4.12. Bevacizumab

Bevacizumab (Avastin, Genentech, Inc., South San Francisco, CA) is a humanized murine antibody approved for the treatment of various cancers and binds all VEGF-A isoforms. The use of bevacizumab for ocular disease has been off-label use [155]. Compared with triamcinolone or laser therapy bevacizumab given every 3-4 weeks showed an increase in visual acuity and reduction in diabetic macular edema at 6 months, 12 months, and 24 months [155]. Another study did not find a significant difference between the groups after six months, but the frequency was every 12 weeks [156, 157].

### 4.13. Ranibizumab

Ranibizumab (Lucentis, Genentech, Inc.) is a humanized monoclonal antibody fragment that that we developed for ocular use from bevacizumab, so it binds to all VEGF-A isoforms with higher affinity. It was thought that the smaller antibody fragment would allow for easy diffusion through the vitreous to get to the retina. It is the first FDA-approved medication to treat diabetic macular edema and is approved for wet AMD, macular edema due to branch and central retinal vein occlusions [155]. Ranibizumab was shown to be most effective when injected every 4 weeks [158]. When given monthly, ranibizumab alone or ranibizumab with laser compared with laser alone showed significant visual acuity improvement and decreased macular edema [159]. At 24 months the ranibizumab and ranibizumab with laser groups showed stable vision, but the laser group showed improved vision [160].

### 4.14. Aflibercept

Aflibercept (Eylea, Regeneron, Tarrytown, NY) is a fusion protein with VEGFR-1 and VEGFR-2 binding domains bound to the Fc of human IgG1. This allows it to bind all isoforms of VEGF-A, VEGF-B, and placental growth factor with high affinity [161]. Aflibercept is approved for wet AMD and macular edema, and a systemic formulation is approved for colorectal cancer. The efficacy of aflibercept has been tested in randomized groups consisting of laser or aflibercept and showed that aflibercept showed greater improvements and no worsening at 52 weeks when given every 4 weeks or 8 weeks [155].

## 5. Future Directions

### 5.1. Delivery of Anti-VEGF as an Intraocular Implant

There are several sustained release intraocular substrates that are in development for the delivery of anti-VEGF neutralization antibodies. The typical polymer matrices used as biodegradable drug delivery systems such as poly(lactic-co-glycolic acid) (PLGA), polylactic acid (PLA), polycaprolactone dimethacrylate (PCM), and polyhydroxyethyl methacrylate (poly-HEMA) have been used as substrates to release bevacizumab for several months [162–164]. Yandrapu et al. demonstrated antibody release in rats for several months with PLGA and PLA nanoparticles, because PLGA is porous and it slowly releases the antibody bound to PLA nanoparticles encased in PLGA [163]. PCM and poly-HEMA have also been used as a sustained release substrate for bevacizumab. This has been demonstrated in rabbits to release antibody for up to 4 months [164]. In addition, there are other polymer matrices waiting to be translated into the clinic. Ranibizumab has been loaded onto microparticles by coaxial electrospray for sustained delivery [165]. The intracellular VEGF signaling cascade is also a target for small molecule inhibitors. Other examples of anti-VEGF treatment are silk hydrogels with other VEGF inhibitors such as a novel single-chain antibody fragment [166, 167].

Neurotech, Inc. (Cumberland, RI), is developing an implant, NT-503 ECT, that can be sutured to the sclera to deliver a continuous supply of anti-VEGF antibody. This is achieved with genetically modified retinal pigmented epithelial (RPE) cells to produce the medication. The modified RPE cells are coated on a polyethylene terephthalate yarn encased in a polysulfone membrane. The polysulfone membrane allows for nourishment of the RPE cells through diffusion of nutrients into the device and the antibody readily diffuses out into the vitreous for over two years [168, 169].

### 5.2. Additional Nonsteroidal Treatment Options

Retinal detachment can be a complication associated with DM. This is due to the ingrowth of retinal vessels into the vitreous that can pull the retina away from the RPE cells as the vitreous moves around. Ocriplasmin (ThromboGenics NV, Iselin, NH) is recombinant human plasmin that digests fibronectin, laminin, and collagen in the vitreous to weaken adhesions at the vitreoretinal surface and release the retinal vessels that have grown into the vitreous. Ocriplasmin has been FDA approved for vitreomacular traction when associated with a macular hole ≤ 400 um [170, 171].

Flt23k intraceptor inhibits VEGF secretion by sequestering it in the endoplasmic reticulum [162]. A Flt23k expression plasmid has been shown to inhibit AMD in primates and murine models [172]. Integrin receptors that are upregulated during ocular neovascularization can be targeted with the RGD peptide motif [173]. By coupling the RGD motif with PLGA nanoparticles and the Flt23k expression plasmid, Luo et al., were able to achieve a targeted gene therapy approach [172].

Another method of inhibiting VEGF expression is through the use of siRNA. It has been demonstrated by Zhang et al. that VEGFR1 siRNA can be effectively delivered with a PEGylated liposome-protamine-hyaluronic acid nanoparticle system to RPE cells. This system was shown to be much more effective at inhibiting a laser induced CNV compared with treatment with naked siRNA [174].

### 5.3. Systemic Therapies/Glucose Monitoring

Risk factors for DR and perhaps other ocular complications include not only hyperglycemia but also hypertension and dyslipidemia [14]. This highlights the fact that glycemic control, as well as other systemic approaches (e.g., blood pressure lowering therapies and lipid lowering therapies), constitutes an important holistic preventative approach to diabetes-related ocular conditions. As far as systemic approaches, stringent glycemic control remains the cornerstone of prevention. Indeed, stringent systemic control of diabetes likely will prevent the progression of DR from the nonproliferative to the proliferative stage and thus prevent the necessity of end-stage treatments such as laser treatment [175]. Tight glucose control is of paramount importance throughout the time course of diabetes in order to most effectively prevent organ complications. For example, studies have shown that poor glucose control in the early stages of diabetes, even if corrected, still may have lasting effects on prognosis of complications long-term [176]. Moreover, glycemic variability is known to associate with adverse diabetes complications [177] and, in type 1 diabetic patients, high glycemic variability predicts microvascular complications [178]. Accordingly, continuous glucose monitoring devices may be a necessary component of an optimal treatment plan.

Of particular relevance to this review and discussed in more detail above, one of the latest experimental approaches to continuous glucose monitoring involves a state-of-the-art contact lens, which noninvasively monitors blood glucose via continuous sensing of glucose in tear fluid. These contact lens-based sensors would then transmit this information to an external device [179, 180].

### 5.4. Adipose-Derived Stem Cells

Unfortunately, in most cases, insulin treatment can only delay the onset and progression of DR but will not prevent or cure the condition [181]. The concept of repairing terminally differentiated organs, such as the eye, with cell-based therapy is gaining traction; such therapy for DR is a promising alternative approach. Intravitreal stem cell injections are an example of a cell-based therapy for DR. Adipose stem cells (ASCs) are a novel cell-based therapy and unique in that they have functional and phenotypic overlap with pericytes which line microvessels in adipose [182, 183]. These particular stem cells also produce angiogenic as well as antiapoptotic factors [184], making them particularly attractive for DR. Importantly, at least one rodent study has shown that a single intravitreal injection of ASCs significantly improved diabetic ocular complications [185].

## 6. Summary

The merging of technologies and explosion of biologics as therapeutics promises to provide additional novel and more effective treatment options as implants or through other delivery methods. Moreover, as the fields of angiogenesis, immunology, and metabolism continue to discover more overlap new pathways will be identified as potential therapeutic targets. Therefore, in the next 5–10 years we anticipate the addition of many new treatments for ocular diabetic complications as exciting discoveries at the bench are translated to the bedside.

## Figures and Tables

**Table 1:** Progressive stages of diabetic retinopathy and the clinical signs.

Stages of diabetic retinopathy	Clinical signs
Mild NPDR	Microaneurysms
Moderate NPDR	Intraretinal hemorrhages, hard exudates, cotton wool spots, and venous beading less than required for severe NPDR
Severe NPDR	Extensive intraretinal hemorrhages in each of 4 quadrants, venous beading in more than 2 quadrants, and one intraretinal microvascular abnormality
PDR	Neovascularization, vitreous/preretinal hemorrhage

NPDR: nonproliferative diabetic retinopathy. PDR: proliferative diabetic retinopathy.

**Table 2:** Clinical manifestations of diabetes mellitus in ocular tissues.

Disease stage	Ocular tissue		
	Cornea	Nerve	Retina
Early/prediabetes	Ocular lens *α*-crystallin formation [60]; increased central corneal thickness [61]	Decreased corneal nerve fiber length [62]	Microaneurysms; exudate; tortuosity of vessels; edema
Midpoint	Reduced corneal subbasal nerve density; increased corneal sensitivity; corneal erosion syndrome [63]	Reduced subfoveal choroidal thickness [64]; reduced corneal nerve density [65]	Macula edema; hemorrhage; retinal detachment
Late-stage diabetes	Reduced tear meniscus parameters [66]; lower nerve fiber and branch length [67]	Optic neuropathy	Severe hemorrhage; retinal tearing/detachment; blindness
